# Frequency of Discordant Documentation of Patient Race and Ethnicity

**DOI:** 10.1001/jamanetworkopen.2024.0549

**Published:** 2024-03-11

**Authors:** Rama A. Salhi, Michelle L. Macy, Margaret E. Samuels-Kalow, Megan Hogikyan, Keith E. Kocher

**Affiliations:** 1 Department of Emergency Medicine, Massachusetts General Hospital, Boston; 2 Division of Emergency Medicine, Department of Pediatrics, Northwestern University Feinberg School of Medicine and Ann & Robert H. Lurie Children’s Hospital of Chicago, Chicago, Illinois; 3 Mary Ann & J. Milburn Smith Child Health Outcomes, Research and Evaluation Center, Stanley Manne Children’s Research Institute, Chicago, Illinois; 4 Department of Emergency Medicine, University of Michigan, Ann Arbor; 5 Department of Learning Health Sciences, University of Michigan, Ann Arbor

## Abstract

This cohort study examines longitudinal changes in race and ethnicity assignment in US hospitals.

## Introduction

The inclusion of race and ethnicity data is critical for assessing equity in medical research, studies of disparities, and national quality measurement in the US.^1,2^ Despite the importance of race and ethnicity to support equity improvement efforts in health care, there remain significant data quality concerns in its collection and reporting in health record systems.^3^ To date, little research has evaluated the reliability of available race and ethnicity data in the emergency department (ED), which serves as an important point of contact for many, often marginalized, patients.^4^ To better understand the magnitude of this issue, we analyzed data from a statewide, multicenter registry of ED visits to evaluate longitudinal changes in race and ethnicity assignment.

## Methods

This retrospective cohort study was conducted on ED visit electronic health record data derived from the Michigan Emergency Department Improvement Collaborative registry, a statewide network of 42 hospitals from 10 health systems, from December 1, 2018, to November 30, 2021. We included patients with at least 2 ED visits to the same hospital or health system in the registry during the study period. Race and ethnicity data were combined into a composite variable: American Indian or Alaska Native, Asian or Pacific Islander (inclusive of Native Hawaiian), Hispanic, non-Hispanic Black (hereafter, Black), non-Hispanic White (hereafter, White), multiracial, or other (races and ethnicities in this category vary among the health care systems and are often reported in aggregate).^5^ For population context, race and ethnicity data of our study sample and census-derived data are provided (eTable in Supplement 1).

The primary outcome of interest was the frequency of discordant documentation of race and ethnicity between ED visits. Descriptive statistics were completed to examine overall trends. Because the collection of race and ethnicity may occur differently for adults than for children, all analyses were stratified accordingly. Analyses were conducted between October 2022 and December 2023 and completed in Stata software, version 17.0 (StataCorp LLC). This study was deemed exempt by the University of Michigan Institutional Review Board because it is secondary research and followed the STROBE reporting guideline.

## Results

Study sample characteristics are outlined in the Table. Of patients with 2 or more ED visits, 6913 of 403 587 adults (1.7%) and 8523 of 121 839 children (7.0%) had discordant racial and ethnic data across visits. Among children, patients categorized as Black or other on the first visit accounted for the largest proportions of discordant documentation (25.8% and 38.7%, respectively) (Figure). Among adults, patients categorized as White, multiracial, or other on their first visit accounted for the largest proportions of discordant documentation (32.3%, 17.7%, and 22.7%, respectively) (Figure).

**Table.** Population Characteristics as Documented on the First ED Visit^a^

Characteristic	Adult		Pediatric	
	Total population (n = 1 191 242)	≥2 ED visits (n = 403 587)	Total population (n = 379 985)	≥2 ED visits (n = 121 839)
Age, median (IQR), y	50 (32-67)	51 (33-69)	6 (1-12)	4 (1-11)
Sex				
Female	551 350 (53.7)	179 868 (55.4)	182 257 (48.0)	59 391 (48.8)
Male	639 513 (46.3)	179 868 (44.6)	197 661 (52.0)	62 429 (51.2)
No. of visits, median (IQR)	1 (1-2)	2 (2-4)	1 (1-2)	2 (2-3)
Racial and ethnic documentation on first ED visit				
American Indian or Alaska Native	3889 (0.3)	1613 (0.4)	943 (0.3)	353 (0.3)
Asian or Pacific Islander	18 634 (1.6)	4666 (1.2)	5107 (1.3)	1308 (1.1)
Black	216 485 (18.2)	89 212 (22.1)	106 125 (27.9)	43 399 (35.6)
Hispanic	51 578 (4.3)	16 746 (4.2)	29 551 (7.8)	10 713 (8.8)
White	842 485 (70.2)	276 664 (68.6)	179 756 (47.3%)	47 979 (39.4%)
Multiracial	8743 (0.7)	3260 (0.8)	7242 (1.9%)	2169 (2.3%)
Other^b^	42 136 (3.5)	10 223 (2.5)	49 174 (12.9%)	14 961 (12.3%)
Missing	0	0	2087 (0.6)	357 (0.3)
Rate of discordance, %	NA	1.7	NA	7.0

Abbreviations: ED, emergency department; NA, not applicable.

^a^ Data are presented as number (percentage) of patients unless otherwise indicated.

^b^ The “other” category is defined at the health system level and reported in aggregate form, limiting detailed description of included categories.

**Figure.**
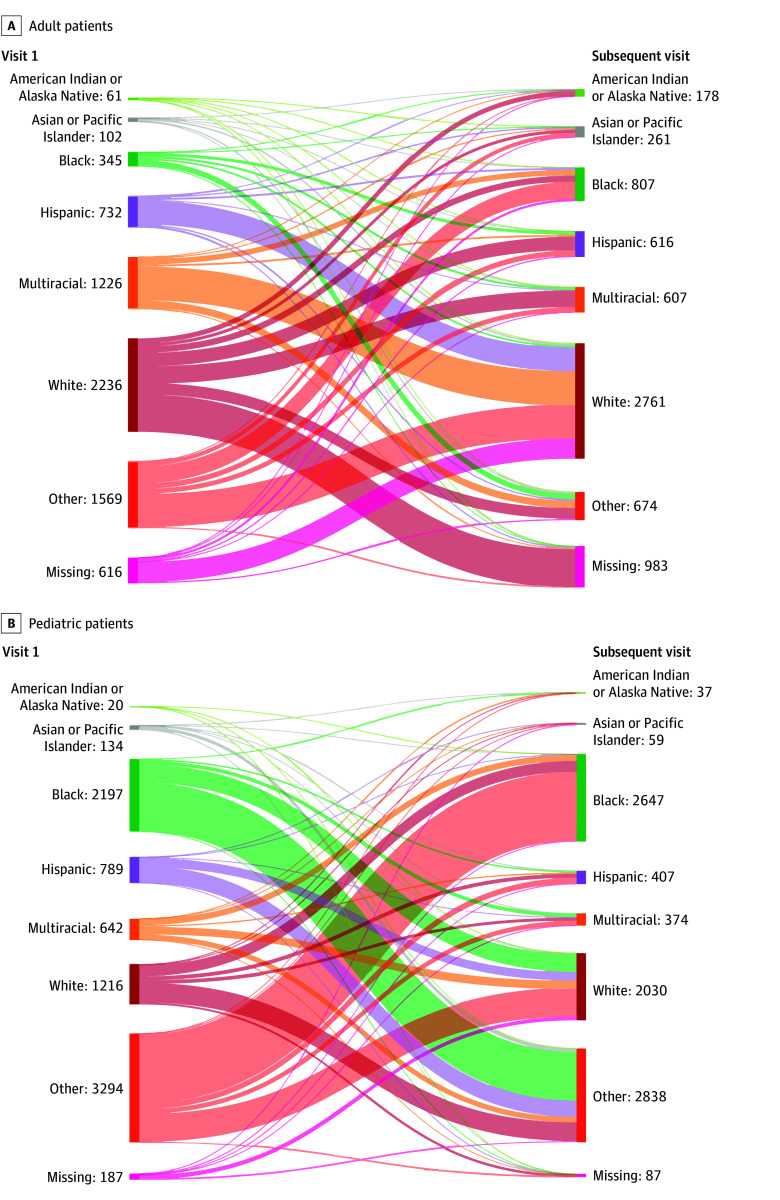
Recorded Race and Ethnicity Among Patients With Discordant Documentation Between Visits The “other” category was defined at the health system level and reported in aggregate form, limiting detailed description of included categories.

## Discussion

Using a large, statewide, multisystem clinical registry, we found significant discordance between ED visits for documentation of race and ethnicity for adults at 1.7% and children at 7.0%. Although we are unable to determine the specific process by which hospitals assign race and ethnicity to their patients, the findings suggest variability and inconsistency in the approach given our focus on repeat ED visits to the same hospital or health care system. Important limitations to consider include reliance on health system record numbers such that cross health system discordance is not captured in our data and true discordance rates may be greater than reported here. Furthermore, given the variability in data collection and reporting practices, rates of self-report as the source of the data are unknown. Finally, because definitions for “other” vary among health care systems and are often reported in aggregate, detailed description of this category is limited.

Our findings shed light on the magnitude of variability that may be introduced into measurement, resource distribution, predictive modeling, policy, and reimbursement, ultimately limiting detection of disparities. Therefore, increased investment in outlining best practices in primary data collection, particularly in the ED, is needed.

## Notes

Supplement 1. **eTable.** Study Sample Demographics in Comparison to State and National Demographics

Supplement 2.
Data Sharing Statement
