# Error in Results

**DOI:** 10.1001/jamanetworkopen.2025.38924

**Published:** 2025-09-24

In the Research Letter titled “Frequency of Discordant Documentation of Patient Race and Ethnicity,”^1^ published March 11, 2024, in the Results section, the outcomes reported in the third and fourth sentences were misattributed as being among adults and children, respectively. The outcomes in the third sentence of the Results apply to children; the outcomes in the fourth sentence apply to adults. This article has been corrected.^1^
