# Supplementary material for: Frequency of Discordant Documentation of Patient Race and Ethnicity
Source: JAMA Netw Open. 2024 Mar 11;7(3):e240549. doi: 10.1001/jamanetworkopen.2024.0549 (PMC10928499; doi:10.1001/jamanetworkopen.2024.0549)
Supplement: Supplement 1. — eTable. Study Sample Demographics in Comparison to State and National Demographics [file jamanetwopen-e240549-s001.pdf]

Supplementary Online Content

Salhi RA, Macy ML, Samuels-Kalow ME, Hogikyan M, Kocher KE. Frequency of discordant documentation of patient race and ethnicity. *JAMA Netw Open*. 2024;7(3):e240549. doi:10.1001/jamanetworkopen.2024.0549

eTable. Study Sample Demographics in Comparison to State and National Demographics

This supplementary material has been provided by the authors to give readers additional information about their work.

© 2024 Salhi RA et al. *JAMA Network Open.*

eTable. Study Sample Demographics in Comparison to State and National Demographics

	STUDY SAMPLE				
	**Total**	**Adult**	**Pediatric**	**Michigan[1]**	**United States[1]**
AMERICAN INDIAN/ALASKA NATIVE	0.3%	0.3%	0.3%	0.7%	1.3%
ASIAN	1.4%	1.5%	1.3%	3.5%	6.3%
BLACK	20.5%	18.2%	27.9%	14.1%	13.6%
HISPANIC	5.2%	4.9%	8.3%	5.7%	19.1%
MULTIRACIAL	1.0%	0.7%	1.9%	2.8%	3.0%
NATIVE HAWAIIAN/OTHER PACIFIC ISLANDER	0.1%	0.1%	0.1%	**	0.3%
WHITE	65.1%	70.7%	47.3%	74.0%	58.9%
OTHER	5.8%	3.5%	12.9%	**	**

[1]United States Census Bureau (2022). Quick Facts. URL: https://www.census.gov/quickfacts

[**]Not Reported

© 2024 Salhi RA et al. *JAMA Network Open.*
