# Supplementary material for: Frequency of Discordant Documentation of Patient Race and Ethnicity
Source: JAMA Netw Open. 2024 Mar 11;7(3):e240549. doi: 10.1001/jamanetworkopen.2024.0549 (PMC10928499; doi:10.1001/jamanetworkopen.2024.0549)
Supplement: Supplement 2. — Data Sharing Statement [file jamanetwopen-e240549-s002.pdf]

Data Sharing Statement

Salhi. Frequency of Discordant Documentation of Patient Race and Ethnicity. *JAMA Netw Open*. Published March 11, 2024. doi:10.1001/jamanetworkopen.2024.0549

Data
Data available: No

Additional Information
Explanation for why data not available: Data can be obtained by request via the process outlined at the Michigan Emergency Department Improvement Collaborative website (https://medicqi.org).
